# Admission NT-proBNP provides stronger prognostic discrimination than the AHEAD score for 1-year mortality in hospitalized acute heart failure: A retrospective cohort study

**DOI:** 10.1371/journal.pone.0353113

**Published:** 2026-07-01

**Authors:** Duc Khanh Nguyen, Thanh Tuan Tran, Van Sy Hoang

**Affiliations:** 1 School of Medicine, University of Medicine and Pharmacy at Ho Chi Minh City (UMP-HCMC), Ho Chi Minh City, Vietnam; 2 Cho Ray Hospital, Ho Chi Minh City, Vietnam; Showa University: Showa Daigaku, JAPAN

## Abstract

**Background:**

Both admission N-terminal pro-B-type natriuretic peptide (NT-proBNP) and the AHEAD score predict prognosis in acute heart failure, but their comparative and complementary value for admission risk stratification remains uncertain.

**Methods:**

We screened 512 consecutive adult hospitalizations for acute heart failure; 478 records had sufficient baseline data, and 430 patients had ascertainable 1-year vital-status follow-up and constituted the analytic cohort. We compared admission NT-proBNP (log-transformed) with the AHEAD score for 1-year all-cause mortality using Cox models, Harrell C-index, apparent calibration, and reclassification (continuous net reclassification improvement [NRI] and integrated discrimination improvement [IDI]). A combined model of both markers and a combined AHEAD x NT-proBNP stratification were also evaluated.

**Results:**

During 1 year, 84 deaths (19.5%) occurred. ln(NT-proBNP) was strongly associated with mortality (adjusted HR 2.63, 95% CI 2.05–3.37 per 1-unit increase; approximately HR 1.95 per doubling). AHEAD categories were associated with mortality in univariable analysis (HR 1.95 for score 2 and 3.61 for score ≥ 3 vs 0−1), but were attenuated after adjustment for ln(NT-proBNP) and admission covariates (adjusted HR 1.03 and 1.81). ln(NT-proBNP) showed higher discrimination than AHEAD categories (Harrell C-index 0.758 vs 0.608). The combined model improved discrimination and reclassification compared with AHEAD alone (C-index 0.757; Delta C-index 0.150; continuous NRI 0.840; IDI 0.136), but not compared with ln(NT-proBNP) alone (Delta C-index 0.000). In combined stratification, the highest NT-proBNP tertile (T3; > 6,385 pg/mL) identified high-risk groups regardless of AHEAD category.

**Conclusions:**

In hospitalized acute heart failure, admission NT-proBNP provided stronger prognostic discrimination than AHEAD categories for 1-year mortality in this cohort. AHEAD may still provide complementary clinical context, but adding AHEAD to ln(NT-proBNP) did not materially improve discrimination beyond NT-proBNP alone. External validation is warranted.

## Background

Acute heart failure (AHF) remains a leading cause of unplanned hospitalization and is associated with substantial mortality despite advances in therapy. Early risk stratification at admission helps guide triage decisions, intensity of monitoring, discharge planning, and timely discussions with patients and families [1–3]. Contemporary guidelines and biomarker-supported care pathways continue to emphasize natriuretic peptides for diagnosis, risk assessment, and post-discharge planning [2,4–6].

N-terminal pro-B-type natriuretic peptide (NT-proBNP) reflects myocardial wall stress and neurohormonal activation and is widely used for diagnosis and risk assessment in heart failure. Admission NT-proBNP has been consistently associated with adverse outcomes across the heart failure spectrum [2,7–9].

Alongside biomarkers, simple clinical scores have been developed to enable rapid classification using routinely available history and comorbidities. The AHEAD score is a parsimonious tool based on atrial fibrillation, anemia, age > 70 years, renal dysfunction, and diabetes mellitus [10,11]. However, clinical scores and biomarkers capture different domains: AHEAD reflects comorbidity burden, whereas NT-proBNP reflects hemodynamic and neurohormonal stress. Their comparative and complementary predictive value may therefore require local validation in diverse health-care systems.

In this retrospective cohort of patients hospitalized with AHF in Vietnam, we evaluated (1) the association of admission NT-proBNP with 1-year all-cause mortality; (2) the association of the AHEAD score with the same outcome; and (3) a clinically interpretable combined admission stratification scheme using AHEAD categories and NT-proBNP strata.

## Methods

### Study design and setting

This retrospective observational cohort study screened 512 consecutive adult patients hospitalized with acute heart failure at a tertiary care center in Vietnam between January 2021 and August 2021.

### Participants

Consecutive admissions with a working diagnosis of acute heart failure were screened. Patients were eligible if acute heart failure was adjudicated as the primary diagnosis for the index hospitalization and if admission NT-proBNP and variables required to calculate the AHEAD score were available. Admissions were excluded when an alternative primary diagnosis dominated the clinical presentation. Of 512 screened patients, 478 records had sufficient baseline data for eligibility assessment and prognostic variables. Among these, 430 patients had ascertainable 1-year vital-status follow-up and constituted the final analytic cohort; 34 records were excluded because baseline data were incomplete and 48 patients were excluded because 1-year vital status could not be ascertained.

### Data collection

Baseline demographics, medical history, vital signs, laboratory values, and echocardiographic parameters obtained at presentation were extracted from medical records. Data were accessed for research purposes on 31/03/2024 (DD/MM/YYYY). Investigators had access to potentially identifiable information during data extraction from the electronic medical record; data were subsequently de-identified before analysis and public sharing. To reduce re-identification risk, exact ages and specific clinical dates are not publicly shared; the public dataset contains only the AHEAD age component (age > 70 years) and relative follow-up/censoring times. NT-proBNP was measured as part of routine admission evaluation in all included patients.

### Exposure definitions

Admission NT-proBNP (pg/mL) was measured as part of routine admission care and was analyzed as a log-transformed continuous variable (natural logarithm, ln[NT-proBNP]). Admission NT-proBNP was selected because the study objective was early risk stratification at hospital presentation, before inpatient treatment, discharge planning, and post-discharge monitoring decisions. Discharge NT-proBNP can provide important residual-risk information but was not measured uniformly in this retrospective cohort; restricting analysis to patients with discharge measurements could introduce selection and survival-to-discharge bias. For descriptive stratification, NT-proBNP was categorized into tertiles using cohort cut points (2,111 and 6,385 pg/mL). The AHEAD score assigned 1 point for each component: atrial fibrillation; anemia (hemoglobin <13 g/dL in men or <12 g/dL in women); age > 70 years; renal dysfunction (serum creatinine >130 micromol/L or documented renal impairment); and diabetes mellitus. The total AHEAD score was grouped a priori as 0–1, 2, and ≥3 to represent low, intermediate, and higher comorbidity burden; this grouping was not derived from tertiles and was selected for clinical interpretability, consistency with prior AHEAD-score applications, and adequate numbers of patients per category [10,11].

### Outcome

The primary outcome was 1-year all-cause mortality. Vital status was ascertained from medical records. Time-to-event was defined as the time from index hospitalization to death. Of 478 patients with sufficient baseline records, 48 had unavailable 1-year vital status and were excluded from the time-to-event analysis. Patients who were alive at 12 months in the analytic cohort were administratively censored at 12 months. In the shared dataset, time_months = 13 denotes event-free follow-up beyond the 12-month endpoint; these observations were analyzed as censored at 12 months.

### Statistical analysis

Continuous variables are presented as mean + /- SD or median (IQR), and categorical variables as counts (percentages). Between-group comparisons used appropriate parametric or non-parametric tests for continuous variables. Categorical variables were compared using chi-square tests when expected cell counts were adequate and Fisher exact tests when sparse cells were present; for 2 x k tables, Fisher-Freeman-Halton exact tests or exact-test extensions were used when appropriate. Kaplan-Meier curves were compared using the log-rank test. Cox proportional hazards models estimated hazard ratios (HRs) with 95% confidence intervals (CIs). Primary models evaluated ln(NT-proBNP) and AHEAD categories. Multivariable models adjusted a priori for sex, systolic blood pressure, heart rate, left ventricular ejection fraction, and serum sodium because these variables represent core demographic, hemodynamic, echocardiographic, and electrolyte markers of admission severity that were not components of the AHEAD score. Renal dysfunction, age, diabetes, anemia, and atrial fibrillation were not added to the primary multivariable model because they are components of the AHEAD score; residual confounding by renal function and treatment variables was addressed in the interpretation and limitations. Analyses used complete-case data for model covariates. No imputation was performed. Missingness was handled by excluding records without complete baseline prognostic variables or 1-year vital status: 34 of 512 screened records had incomplete baseline data and 48 of 478 otherwise eligible records lacked ascertainable 1-year vital status. Two-sided p < 0.05 indicated statistical significance. Analyses were performed using Python (pandas, scipy, statsmodels, scikit-learn, and matplotlib). Because there were no deaths in the lowest NT-proBNP tertile, tertile categories were used for descriptive Kaplan-Meier visualization only and were not used to estimate hazard ratios; categorical analyses therefore focused on the pragmatic combined strata (T1-2 vs T3).

Model discrimination was assessed primarily using Harrell C-index from Cox models [12,13]. We compared the combined model (AHEAD categories + ln[NT-proBNP]) against AHEAD alone and ln(NT-proBNP) alone by estimating bootstrap 95% CIs for differences in C-index (1,000 resamples). ROC AUC analyses were removed from the main manuscript in response to the Editor’s comment and retained only as supplementary sensitivity analyses in S1 Text. Reclassification was evaluated using continuous (category-free) NRI and IDI at 1 year based on predicted probabilities from logistic models, with bootstrap 95% CIs and two-sided p-values. Apparent calibration of the combined model was examined graphically by plotting observed 1-year mortality against mean predicted risk across deciles of predicted risk.

### Pragmatic combined stratification

For clinical interpretability, combined strata were created by crossing AHEAD (0–1 vs ≥ 2) with NT-proBNP (T1-2 vs T3). Cox models used the group AHEAD 0–1 & NT-proBNP T1-2 as the reference. An adjusted model included sex, systolic blood pressure, heart rate, ejection fraction, and sodium.

### Ethics

The study was approved by the Ethics Committee in Biomedical Research, University of Medicine and Pharmacy at Ho Chi Minh City (Approval No. 953; 16 October 2023). Informed consent was waived because this was a retrospective analysis of de-identified data collected as part of routine clinical care, and no intervention was performed.

### Reporting

Reporting follows STROBE guidance for observational studies and, where applicable, TRIPOD principles for prognostic analyses [14].

## Results

Of 512 screened patients, 478 records had sufficient baseline data and 430 patients had ascertainable 1-year vital-status follow-up and constituted the analytic cohort. The remaining 34 screened records had incomplete baseline data, and 48 otherwise eligible records lacked ascertainable 1-year vital status. Baseline characteristics stratified by AHEAD categories are shown in Table 1. Patients with higher AHEAD scores had a higher prevalence of the age > 70 years component and other AHEAD-score components by definition; they also had higher NT-proBNP and creatinine values and lower hemoglobin values. AHEAD components are reported descriptively because they define the AHEAD score categories.

**Table 1:** Baseline characteristics stratified by AHEAD categories.

Variable	Overall (n = 430)	AHEAD 0–1 (n = 97)	AHEAD 2 (n = 113)	AHEAD >=3 (n = 220)	P value
Male sex, n (%)	244 (56.7%)	74 (76.3%)	64 (56.6%)	106 (48.2%)	<0.001
Systolic blood pressure, mmHg	108.7 ± 22.1	107.7 ± 25.8	107.1 ± 25.3	109.9 ± 18.2	.480
Heart rate, beats/min	97.5 ± 15.7	95.5 ± 12.4	98.3 ± 14.8	98.0 ± 17.3	.367
Ejection fraction, %	43.7 ± 6.1	44.6 ± 5.9	42.9 ± 6.8	43.7 ± 5.7	.117
NT-proBNP, pg/mL	3774 (1633-8269)	1717 (1390-3718)	4234 (1580-10499)	4912 (2410-9784)	<0.001
Creatinine, mg/dL	1.28 (1.11-1.53)	1.16 (1.03-1.27)	1.22 (1.02-1.39)	1.40 (1.22-1.68)	<0.001
Hemoglobin, g/dL	12.1 ± 2.4	14.1 ± 1.9	13.0 ± 1.7	10.8 ± 2.0	<0.001
Sodium, mmol/L	136.6 ± 4.8	137.6 ± 4.5	136.1 ± 5.8	136.4 ± 4.4	.064
Atrial fibrillation component, n (%)	73 (17.0%)	2 (2.1%)	21 (18.6%)	50 (22.7%)	<0.001
Anemia component, n (%)	244 (56.7%)	20 (20.6%)	33 (29.2%)	191 (86.8%)	<0.001
Renal dysfunction component, n (%)	333 (77.4%)	39 (40.2%)	85 (75.2%)	209 (95.0%)	<0.001
Diabetes mellitus component, n (%)	124 (28.8%)	6 (6.2%)	17 (15.0%)	101 (45.9%)	<0.001
Age > 70 years component, n (%)	268 (62.3%)	6 (6.2%)	70 (61.9%)	192 (87.3%)	<0.001

Values are mean + /- SD, median (IQR), or n (%). AHEAD components are shown descriptively because they define the AHEAD categories. P values are from ANOVA or Kruskal-Wallis tests for continuous variables and exact/chi-square tests for categorical variables, as appropriate. Exact age values are not reported in Table 1 or included in the public dataset; the age > 70 years component is shown because it is required to reproduce the AHEAD score.

**Fig 1:**
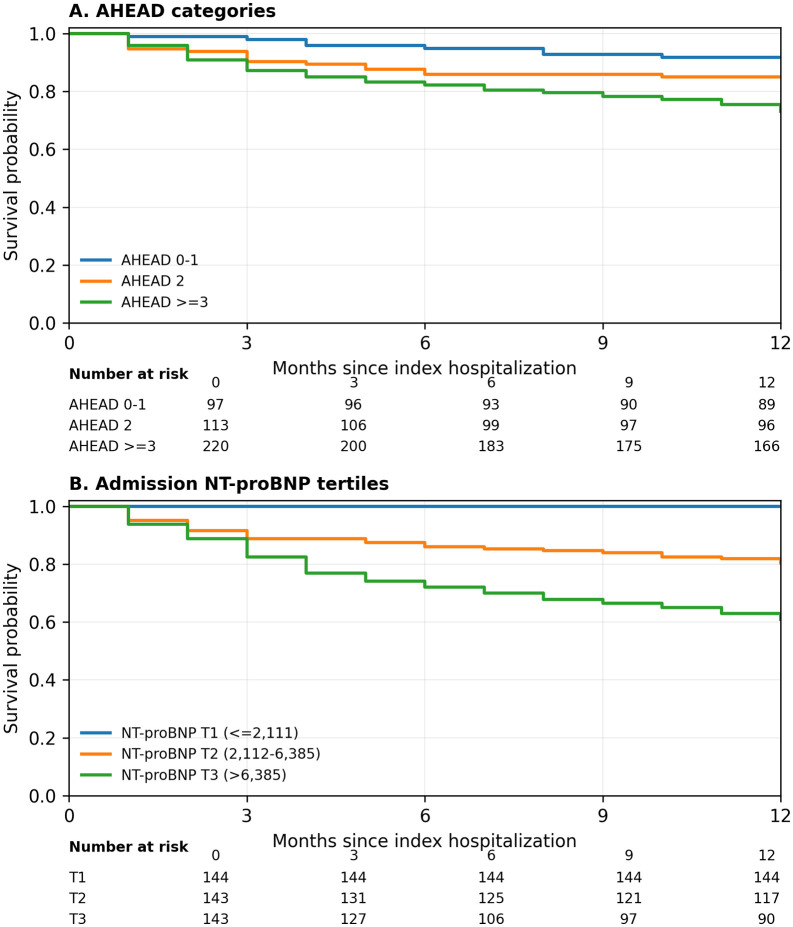
Kaplan-Meier survival curves by AHEAD category and admission NT-proBNP tertile. (A) Kaplan-Meier estimates of 1-year all-cause survival stratified by AHEAD category (0−1, 2, and ≥3). (B) Kaplan-Meier estimates of 1-year all-cause survival stratified by admission NT-proBNP tertile using cohort cut points of 2,111 and 6,385 pg/mL (T1 ≤ 2,111; T2 2,112−6,385; T3 > 6,385 pg/mL). For both panels, time-to-event was defined as months from the index hospitalization to death; survivors were administratively censored at 12 months, and numbers at risk are displayed below the plots. Alt text: Two Kaplan-Meier plots showing 1-year survival after hospitalization for acute heart failure, stratified in panel A by AHEAD category and in panel B by admission NT-proBNP tertile.

During 1 year of follow-up, 84 deaths (19.5%) occurred among the 430 analytic patients; 346 patients were alive and administratively censored at 12 months. Deaths occurred in 8.2%, 15.0%, and 26.8% of patients in the AHEAD 0–1, AHEAD 2, and AHEAD ≥ 3 groups, respectively. By NT-proBNP tertiles, mortality was 0.0%, 19.6%, and 39.2% in T1, T2, and T3, respectively. Kaplan-Meier curves demonstrated stepwise separation by AHEAD category and a more pronounced separation by admission NT-proBNP tertiles, including no deaths in the lowest tertile (Fig 1). Given the absence of deaths in the lowest tertile, we did not estimate hazard ratios across NT-proBNP tertiles.

In Cox models, ln(NT-proBNP) was strongly associated with 1-year mortality in univariable analysis (HR 2.48, 95% CI 1.98–3.11; p < 0.001) and remained independently associated after multivariable adjustment (HR 2.63, 95% CI 2.05–3.37; p < 0.001), corresponding to an approximate 1.95-fold higher hazard per doubling of NT-proBNP. AHEAD score ≥ 3 was associated with mortality in univariable analysis, but AHEAD categories were attenuated after inclusion of ln(NT-proBNP) and admission covariates (Table 2).

**Table 2:** Cox regression models for 1-year all-cause mortality (ln[NT-proBNP] and AHEAD categories).

Predictor	Univariable HR (95% CI)	P (uni)	Multivariable HR (95% CI)	P (multi)
ln(NT-proBNP) (per 1-unit increase)	2.48 (1.98-3.11)	<0.001	2.63 (2.05-3.37)	<0.001
AHEAD 2 vs 0–1	1.95 (0.84-4.51)	.121	1.03 (0.43-2.45)	.945
AHEAD >=3 vs 0–1	3.61 (1.73-7.56)	<0.001	1.81 (0.83-3.92)	.135

*Multivariable HR for ln(NT-proBNP) is adjusted for sex, systolic blood pressure, heart rate, left ventricular ejection fraction, and serum sodium. Multivariable HRs for AHEAD categories are adjusted for ln(NT-proBNP) plus the same covariates. NT-proBNP tertiles are presented descriptively in Kaplan-Meier analyses; no HRs were estimated across tertiles because there were no deaths in the lowest tertile.*

**Fig 2:**
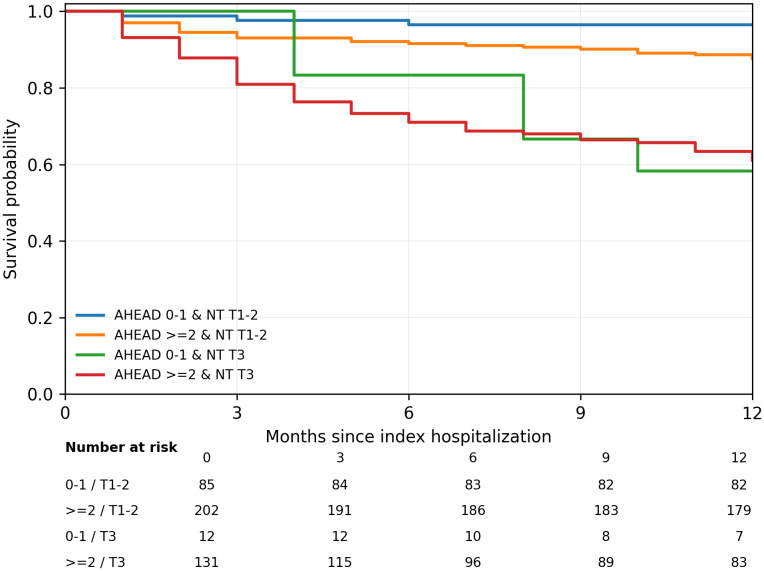
Survival by combined AHEAD and NT-proBNP strata. Kaplan-Meier estimates of 1-year all-cause survival using four admission strata defined by AHEAD category (0-1 vs ≥ 2) and NT-proBNP (T1-2 vs T3): AHEAD 0-1 & NT-proBNP T1-2, AHEAD ≥ 2 & NT-proBNP T1-2, AHEAD 0-1 & NT-proBNP T3, and AHEAD ≥ 2 & NT-proBNP T3. Time-to-event was defined as months from the index hospitalization to death; survivors were administratively censored at 12 months, and numbers at risk are displayed below the plot. In the figure, NT denotes NT-proBNP. Alt text: Kaplan-Meier plot showing 1-year survival after hospitalization for acute heart failure across four combined AHEAD and NT-proBNP strata.

In the pragmatic combined stratification, NT-proBNP T3 identified high-risk groups regardless of AHEAD category. Compared with the reference group (AHEAD 0–1 & NT-proBNP T1-2), adjusted HRs were 14.02 (95% CI 3.26–60.27) for AHEAD 0–1 & NT-proBNP T3 and 14.30 (95% CI 4.38–46.61) for AHEAD ≥ 2 & NT-proBNP T3 (both p < 0.001). AHEAD ≥ 2 with NT-proBNP T1-2 also showed elevated risk but with a lower HR than the T3 groups (adjusted HR 3.67, 95% CI 1.10–12.21; p = 0.034) (Table 3; Fig 2).

**Table 3:** Cox regression models for combined AHEAD and NT-proBNP strata.

Combined group (ref: AHEAD 0–1 & NT-proBNP T1-2)	Univariable HR (95% CI)	P (uni)	Multivariable HR (95% CI)	P (multi)
AHEAD 0–1 & NT-proBNP T3	13.37 (3.19-55.99)	<0.001	14.02 (3.26-60.27)	<0.001
AHEAD >=2 & NT-proBNP T1-2	3.68 (1.11-12.17)	.033	3.67 (1.10-12.21)	.034
AHEAD >=2 & NT-proBNP T3	13.59 (4.24-43.56)	<0.001	14.30 (4.38-46.61)	<0.001

**Fig 3:**
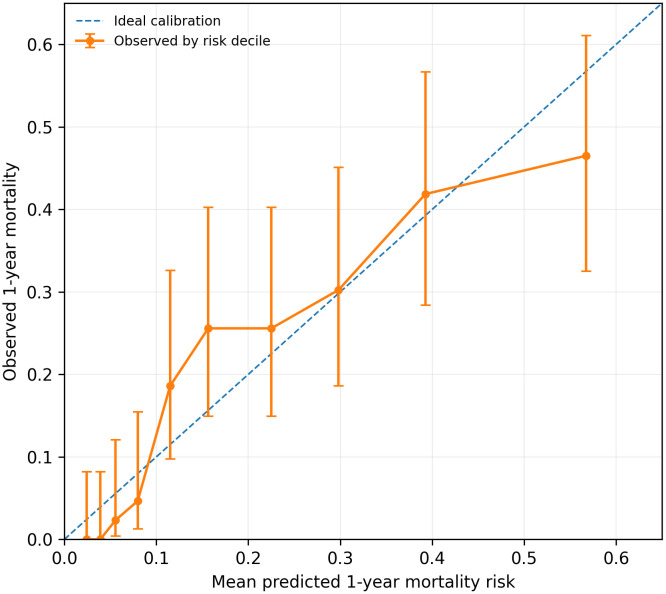
Apparent calibration of the combined prognostic model. Observed 1-year all-cause mortality is plotted against mean predicted 1-year mortality across deciles of predicted risk from the model including AHEAD categories and ln(NT-proBNP). The dashed diagonal line indicates ideal calibration; vertical bars show 95% confidence intervals for observed event proportions. Alt text: Calibration plot comparing observed and predicted 1-year mortality across ten risk deciles for the combined AHEAD plus ln(NT-proBNP) model.

In discrimination analyses, ln(NT-proBNP) provided substantially higher discrimination than AHEAD categories (Harrell C-index 0.758 vs 0.608). The combined model achieved a C-index of 0.757. Compared with AHEAD alone, the combined model significantly improved discrimination (Delta C-index 0.150, 95% CI 0.101–0.203; p < 0.001) and yielded significant reclassification (continuous NRI 0.840, 95% CI 0.634–1.047; IDI 0.136, 95% CI 0.098–0.178; both p < 0.001). Adding AHEAD categories to ln(NT-proBNP) did not materially improve discrimination (Delta C-index 0.000, 95% CI −0.017 to 0.016; p = 0.956), although reclassification indices suggested modest changes (continuous NRI 0.474, 95% CI 0.239–0.690; IDI 0.017, 95% CI 0.002–0.031) (Tables 4 and 5). AUC results are reported as supplementary sensitivity analyses in S1 Text. In apparent calibration analysis, observed 1-year mortality across deciles of predicted risk generally increased with predicted risk, although calibration was imperfect in some deciles (Fig 3).

**Table 4:** Model discrimination for 1-year mortality.

Model	Predictors	Harrell C-index (95% CI)
AHEAD categories	AHEAD 2 vs 0–1; AHEAD ≥ 3 vs 0–1	0.608 (0.553-0.658)
ln(NT-proBNP)	ln(NT-proBNP) at admission	0.758 (0.712-0.804)
Combined	AHEAD categories + ln(NT-proBNP)	0.757 (0.713-0.803)

**Table 5:** Incremental value of the combined model.

Comparison	Delta C-index (95% CI), p	Continuous NRI / IDI (95% CI), p
Combined vs AHEAD	0.150 (0.101–0.203), p < 0.001	NRI 0.840 (0.634–1.047), p < 0.001; IDI 0.136 (0.098–0.178), p < 0.001
Combined vs ln(NT-proBNP)	0.000 (−0.017–0.016), p = .956	NRI 0.474 (0.239–0.690), p < 0.001; IDI 0.017 (0.002–0.031), p = .024

AUC analyses were removed from the main manuscript and are reported only as supplementary sensitivity analyses in S1 Text.

## Discussion

In this real-world cohort of patients hospitalized with AHF, admission NT-proBNP demonstrated a strong and independent association with 1-year all-cause mortality. When NT-proBNP was modeled as a continuous log-transformed variable, it remained robust after adjustment for key admission covariates, supporting its role as a pragmatic biomarker for early prognostic risk stratification.

The results should be interpreted as a predictive comparison rather than a causal or clinical superiority claim. NT-proBNP is a biomarker of hemodynamic stress and disease severity, whereas AHEAD is a simple comorbidity-based clinical score. ln(NT-proBNP) had better discrimination than AHEAD categories; however, AHEAD still provided clinical context and combined stratification identified a gradient in which very high NT-proBNP was associated with the highest risk while elevated AHEAD score with lower NT-proBNP carried intermediate risk. Thus, the two approaches are complementary rather than mutually exclusive.

These results have practical implications for admission workflows. We do not propose replacing bedside assessment or comorbidity-based scores with NT-proBNP alone. Instead, a routinely available admission biomarker, interpreted alongside a brief clinical score, may support rapid triage, early senior review, escalation of monitoring, discharge planning, and structured communication of prognosis. In settings where NT-proBNP testing is available, patients in the highest NT-proBNP tertile may warrant closer monitoring and earlier follow-up planning, while the AHEAD score provides complementary information about comorbidity burden. Formal implementation should await external validation and clinical-utility analyses.

This study has limitations. First, the single-center design and retrospective data abstraction may limit generalizability and introduce residual confounding. Second, NT-proBNP is influenced by renal function and disease severity. We did not include renal dysfunction in the primary multivariable model because it is a component of AHEAD, and treatment variables were not uniformly available; therefore, residual confounding remains possible. Third, the absence of deaths in the lowest NT-proBNP tertile implies limited events for some strata, widening confidence intervals for group comparisons. Fourth, complete-case analysis was used: 34 screened records had incomplete baseline data and 48 otherwise eligible records lacked ascertainable 1-year vital status. Excluding these patients may introduce bias if missingness was not random. Fifth, discharge NT-proBNP was not measured uniformly; this study therefore addresses admission risk stratification rather than residual risk at discharge. Finally, we performed internal comparisons of discrimination, continuous reclassification, and apparent calibration, but we did not undertake external validation or formal clinical-utility analyses (e.g., decision-curve analysis); therefore, performance estimates may be optimistic and should be confirmed in independent cohorts.

Future studies should assess external calibration, discrimination, and clinical utility across diverse populations and evaluate whether incorporating admission NT-proBNP into structured clinical pathways improves patient-centered outcomes and resource allocation.

## Conclusions

Admission NT-proBNP is a robust predictor of 1-year mortality among patients hospitalized with AHF and provides stronger prognostic discrimination than AHEAD categories in this cohort. AHEAD may still provide complementary clinical context, but adding AHEAD categories to ln(NT-proBNP) did not materially improve discrimination beyond ln(NT-proBNP) alone. A pragmatic combined scheme using NT-proBNP with a brief clinical score may support early admission risk stratification; external validation is warranted.

## Supporting information

S1 Text Supplementary discrimination and reclassification analyses for 1-year mortality (C-index/AUC/NRI/IDI). (DOCX)

S1 Checklist STROBE checklist for cohort studies. (DOCX)

## Abbreviations

AHF: acute heart failure
NT-proBNP: N-terminal pro-B-type natriuretic peptide
HR: hazard ratio
CI: confidence interval
SBP: systolic blood pressure
LVEF: left ventricular ejection fraction
IQR: interquartile range
NRI: net reclassification improvement
IDI: integrated discrimination improvement
