# Supplementary material for: Admission NT-proBNP provides stronger prognostic discrimination than the AHEAD score for 1-year mortality in hospitalized acute heart failure: A retrospective cohort study
Source: PLoS One. 2026 Jul 1;21(7):e0353113. doi: 10.1371/journal.pone.0353113 (PMC13322554; doi:10.1371/journal.pone.0353113)
Supplement: S1 Text — (DOCX) [file pone.0353113.s001.docx]

# S1 Text. Supplementary discrimination and reclassification analyses for 1-year mortality (C-index/AUC/NRI/IDI).

This supporting information provides additional details of discrimination and reclassification analyses comparing admission NT-proBNP with the AHEAD score for predicting 1-year all-cause mortality in hospitalized acute heart failure.

## Methods

We evaluated three prognostic models: (i) AHEAD score categories (0-1 as reference; 2; >=3), (ii) ln-transformed admission NT-proBNP, and (iii) the combined model including AHEAD categories plus ln(NT-proBNP). Model associations with time-to-death were estimated using Cox proportional hazards regression.

Discrimination was quantified using Harrell C-index as the main discrimination measure and the area under the receiver operating characteristic curve (AUC) for 1-year mortality as a supplementary sensitivity analysis based on predicted 1-year risk. Uncertainty intervals were obtained using bootstrap resampling (1,000 replicates).

Incremental predictive value of the combined model versus AHEAD alone and versus ln(NT-proBNP) alone was assessed by differences in C-index and AUC, and by continuous NRI and IDI, with 95% bootstrap confidence intervals and two-sided p-values.

## Results

As shown in Table A in S1 Text, ln(NT-proBNP) demonstrated substantially higher discrimination than AHEAD categories. Adding AHEAD categories to ln(NT-proBNP) did not materially change C-index or AUC, although continuous reclassification indices suggested modest changes.

**Table A. Model discrimination.**

| **Model** | **Predictors** | **Harrell C-index (95% CI)** | **AUC for 1-year death (95% CI)** |
| --- | --- | --- | --- |
| AHEAD categories | AHEAD 2 vs 0-1; AHEAD >=3 vs 0-1 | 0.608 (0.553-0.658) | 0.631 (0.575-0.684) |
| ln(NT-proBNP) | ln(NT-proBNP) at admission | 0.758 (0.712-0.804) | 0.793 (0.750-0.840) |
| Combined | AHEAD categories + ln(NT-proBNP) | 0.757 (0.713-0.803) | 0.797 (0.753-0.842) |

**Table B. Incremental value of the combined model.**

| **Comparison** | **Delta C-index (95% CI), p** | **Delta AUC (95% CI), p** | **Continuous NRI / IDI (95% CI), p** |
| --- | --- | --- | --- |
| Combined vs AHEAD | 0.150 (0.101-0.203), p<0.001 | 0.166 (0.114-0.222), p<0.001 | NRI 0.840 (0.634-1.047), p<0.001; IDI 0.136 (0.098-0.178), p<0.001 |
| Combined vs ln(NT-proBNP) | 0.000 (-0.017-0.016), p=.956 | 0.004 (-0.013-0.021), p=.694 | NRI 0.474 (0.239-0.690), p<0.001; IDI 0.017 (0.002-0.031), p=.024 |

Abbreviations: AUC, area under the receiver operating characteristic curve; CI, confidence interval; IDI, integrated discrimination improvement; NRI, net reclassification improvement; NT-proBNP, N-terminal pro-B-type natriuretic peptide.

Notes: Confidence intervals were obtained by bootstrap resampling (1,000 replicates).
