# Supplementary material for: Admission NT-proBNP provides stronger prognostic discrimination than the AHEAD score for 1-year mortality in hospitalized acute heart failure: A retrospective cohort study
Source: PLoS One. 2026 Jul 1;21(7):e0353113. doi: 10.1371/journal.pone.0353113 (PMC13322554; doi:10.1371/journal.pone.0353113)
Supplement: S1 Checklist — (DOCX) [file pone.0353113.s002.docx]

# STROBE Statement-Checklist of items that should be included in reports of cohort studies

Manuscript: Admission NT-proBNP vs AHEAD score for 1-year mortality in acute heart failure (PLOS ONE resubmission)

Corresponding author: Duc Khanh Nguyen

| **Section/Item** | **Recommendation** | **Reported in revised manuscript** |
| --- | --- | --- |
| Title/Abstract 1a | Indicate the study design with a commonly used term in the title or abstract. | Title; Abstract |
| Abstract 1b | Provide an informative and balanced summary of what was done and found. | Abstract |
| Background/rationale 2 | Explain the scientific background and rationale. | Background |
| Objectives 3 | State specific objectives. | Background, final paragraph |
| Study design 4 | Present key elements of study design early. | Methods, Study design and setting |
| Setting 5 | Describe setting, dates, recruitment/exposure/follow-up/data collection. | Methods, Study design and setting; Data collection |
| Participants 6a | Give eligibility criteria and methods of selection/follow-up. | Methods, Participants; Outcome |
| Participants 6b | Matched studies: give matching criteria. | N/A |
| Variables 7 | Define outcomes, exposures, predictors, confounders. | Methods, Exposure definitions; Outcome; Statistical analysis |
| Data sources/measurement 8 | Give sources of data and methods of assessment. | Methods, Data collection |
| Bias 9 | Describe efforts to address potential sources of bias. | Methods; Discussion, Limitations |
| Study size 10 | Explain how study size was arrived at. | Methods, Participants |
| Quantitative variables 11 | Explain how quantitative variables were handled. | Methods, Exposure definitions; Statistical analysis |
| Statistical methods 12a | Describe statistical methods including confounding control. | Methods, Statistical analysis |
| Statistical methods 12b | Describe subgroup/interactions if applicable. | Methods, Pragmatic combined stratification |
| Statistical methods 12c | Explain how missing data were addressed. | Complete-case analytic cohort; 34 records excluded for incomplete baseline data; 48 excluded for unavailable 1-year vital status. |
| Statistical methods 12d | Explain how loss to follow-up was addressed. | 48 otherwise eligible patients lacked ascertainable 1-year vital status; 430 patients included in time-to-event analysis. |
| Statistical methods 12e | Describe sensitivity analyses. | S1 Text (AUC as supplementary sensitivity analysis). |
| Participants 13a | Report numbers at each stage. | Results, first paragraph |
| Participants 13b | Give reasons for non-participation. | Results, first paragraph |
| Participants 13c | Consider use of a flow diagram. | Not used; numbers reported in text. |
| Descriptive data 14a | Give characteristics of participants and exposures/confounders. | Table 1 |
| Descriptive data 14b | Indicate missing data for variables. | Methods and Results; analytic dataset complete after exclusions. |
| Outcome data 15 | Report outcome events over time. | Results; Figures 1-2 |
| Main results 16a | Give unadjusted and adjusted estimates with precision. | Results; Tables 4 and 5; S1 Text |
| Main results 16b | Report category boundaries. | Methods; Table/Figure legends |
| Main results 16c | Translate estimates into absolute risk if relevant. | Results: event rates by AHEAD and NT-proBNP strata. |
| Other analyses 17 | Report other analyses and sensitivity analyses. | Results; Tables 4A and 4B; S1 Text |
| Key results 18 | Summarise key results. | Discussion |
| Limitations 19 | Discuss limitations and potential bias/imprecision. | Discussion, Limitations |
| Interpretation 20 | Give cautious interpretation. | Discussion; Conclusions |
| Generalisability 21 | Discuss generalisability. | Discussion, Limitations |
| Funding 22 | Give source of funding and role of funders. | Financial Disclosure statement in the submission system. |
